# Association Between Sleep-Related Medication Use and Recurrent Falls Among Inpatients: A Five-Year Retrospective Observational Study at a University Hospital

**DOI:** 10.3390/medsci14060693

**Published:** 2026-10-21

**Authors:** Norio Imai, Daisuke Homma, Yugo Shobugawa, Koji Sato, Yoji Horigome, Hiroyuki Kawashima

**Affiliations:** 1 Division of Comprehensive Musculoskeletal Medicine, Graduate School of Medicine, Dentistry and Health Sciences, Niigata University, Niigata 951-8510, Japan; 2 Division of Orthopedic Surgery, Department of Regenerative and Transplant Medicine, Graduate School of Medical and Dental Sciences, Niigata University, Niigata 951-8510, Japan; 3 Institute of Future Science, Institute of Science Tokyo, 1-5-45, Yushima, Tokyo 113-8510, Japan

**Keywords:** inpatient falls, recurrent falls, hypnotics, benzodiazepines, polypharmacy

## Abstract

Objective: The aim of this study is to examine associations between sleep-related medication use at the first inpatient fall and recurrent falls, focusing on drug classes and concurrent use of multiple classes. Methods: We conducted a single-center retrospective observational study of 2587 inpatient fall events involving 1830 patients at our hospital between January 2021 and December 2025. Patients were classified as single fallers (one fall) or recurrent fallers (two or more falls). At the first fall, we assessed benzodiazepine use for insomnia or anxiety, non-benzodiazepine hypnotics (Z-drugs), orexin receptor antagonists, and ramelteon. Modified Poisson regression with a log link and a robust sandwich variance estimator was used to estimate adjusted risk ratios (RRs), with adjustment for age, sex, activity at the first fall, dementia, and total medication count. Results: There were 1394 single fallers and 436 recurrent fallers. In Model 1, adjusted for age, sex, and activity, benzodiazepines (adjusted RR, 1.56; 95% confidence interval [CI], 1.25–1.95), Z-drugs (RR, 1.49; 95% CI, 1.13–1.95), and orexin receptor antagonists (RR, 1.39; 95% CI, 1.16–1.66) were associated with recurrent falls. These associations persisted in Model 2 after additional adjustment for dementia and total medication count: benzodiazepines (RR, 1.54; 95% CI, 1.22–1.94), Z-drugs (RR, 1.46; 95% CI, 1.11–1.92), and orexin receptor antagonists (RR, 1.37; 95% CI, 1.14–1.64). Ramelteon was not significantly associated with recurrent falls (RR, 1.29; 95% CI, 0.96–1.74). Any sleep-related medication use (RR, 1.53; 95% CI, 1.30–1.82) and concurrent use of two or more classes (RR, 1.83; 95% CI, 1.46–2.29) were associated with recurrent falls. Conclusions: Sleep-related medication use, particularly concurrent use of multiple classes, was associated with recurrent inpatient falls. Sleep-related medications should be reviewed at hospital admission and reassessed after an index fall, with particular attention to their indications and concurrent use.

## 1. Introduction

Inpatient falls are among the most common adverse events occurring in hospitalized patients [1,2]. In addition to causing direct physical injury, falls may lead to reduced mobility and activities of daily living, prolonged hospitalization, and changes in discharge destination [3,4]. Furthermore, patients who experience an initial fall may subsequently fall again, making the prevention of recurrent falls after an initial event an important clinical concern [5,6].

Inpatient falls are influenced by numerous factors, including age, cognitive function, mobility, the underlying reason for hospitalization, environmental conditions, and toileting activities [1,2,7]. Among these factors, medication exposure is of particular clinical importance because it is potentially modifiable. In particular, medications acting on the central nervous system may contribute to falls through somnolence, impaired attention, prolonged reaction time, reduced muscle tone, and impaired balance [8,9,10].

Within this broad group of medications, sleep-related medications warrant specific consideration because their pharmacological properties differ substantially. In addition to conventionally used benzodiazepines and non-benzodiazepine hypnotics (Z-drugs), orexin receptor antagonists and ramelteon, a melatonin receptor agonist, have increasingly been used in recent years [11,12]. Because these medications differ in their mechanisms of action and elimination half-lives, their associations with falls may also differ [11,12]. Moreover, some patients receive multiple classes of sleep-related medications concurrently, raising concerns regarding the potential effects of overlapping fall-related medications [13,14].

Despite these pharmacological differences, evidence regarding recurrent inpatient falls remains limited. Although numerous studies have investigated the association between sleep-related medications and the occurrence of falls [10], few have examined individual medication classes and their concurrent use from the perspective of whether patients who have already experienced an inpatient fall subsequently develop recurrent falls [5,6]. Therefore, this study aimed to clarify the association between sleep-related medication use at the time of the first fall and recurrent inpatient falls among patients who experienced inpatient falls during a five-year period.

## 2. Materials and Methods

### 2.1. Study Design and Participants

**Figure 1:**
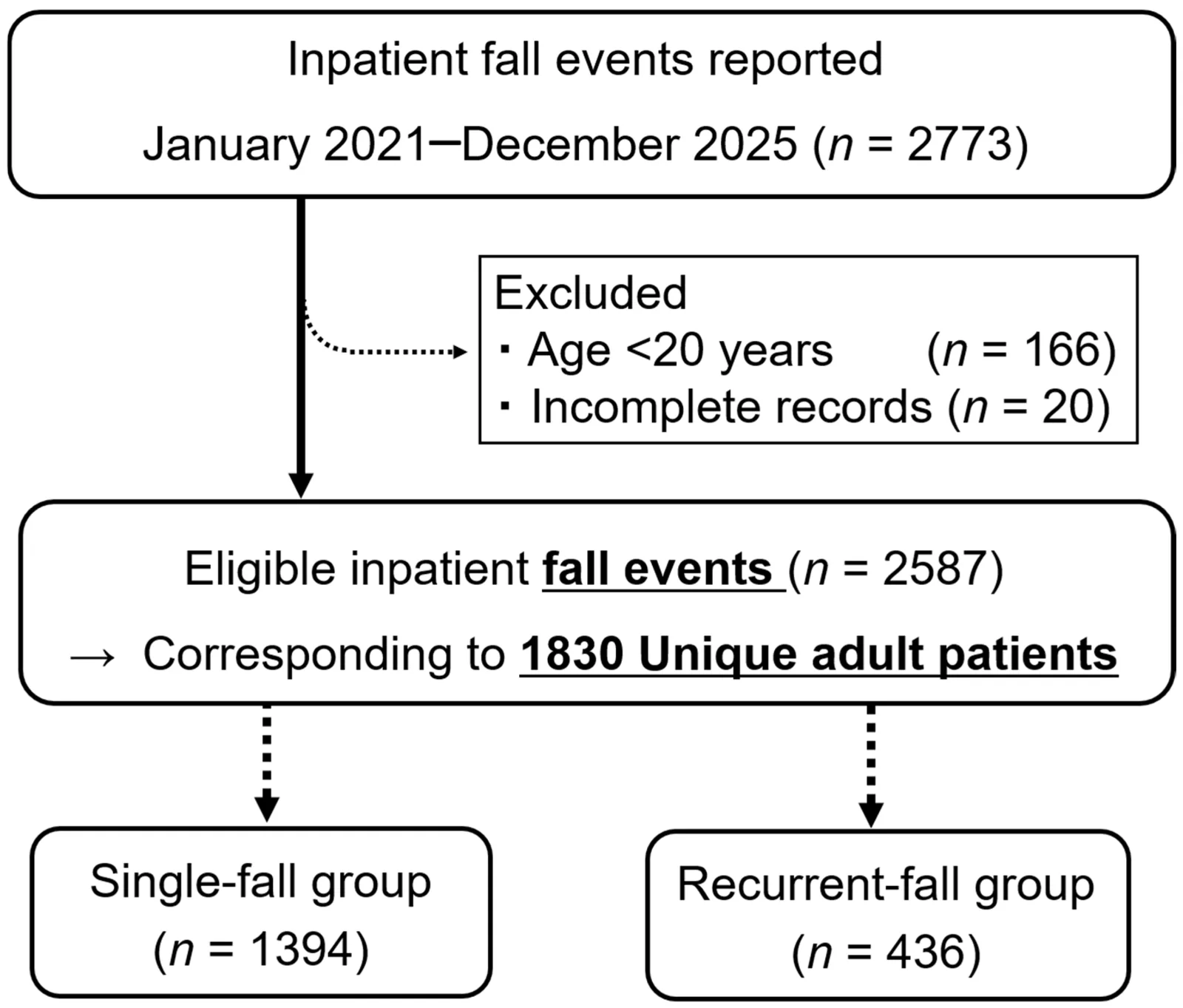
Flowchart of patient selection. Arrows indicate the flow of patient selection and classification into the single-faller and recurrent-faller groups.

This retrospective observational study was conducted at Niigata University Medical and Dental Hospital. We reviewed medical records and incident reports of falls that occurred in the hospital between 1 January 2021 and 31 December 2025. A total of 2773 inpatient fall events were identified during the study period. We excluded 166 events involving patients younger than 20 years and 20 events with incomplete records. Consequently, 2587 inpatient fall events involving 1830 unique patients were included in the study (Figure 1).

Patients were classified as single fallers if they experienced one fall and as recurrent fallers if they experienced two or more falls during the study period.

Patients with only one documented fall during the study period were classified as single fallers, whereas those with two or more documented falls were classified as recurrent fallers. For recurrent fallers, the earliest documented fall was defined as the index fall, and patient characteristics and medication use at the time of the index fall were included in the analysis.

Most participants in the present study overlapped with those in a previous study conducted at the same institution during the same study period, which examined the general characteristics of inpatient falls and factors associated with fall-related fractures and recurrent falls [15]. While the previous study evaluated the use of central nervous system depressants as a broad category, the present study used newly collected and classified medication data to examine the associations of individual sleep-related medication classes—including benzodiazepines prescribed for insomnia or anxiety, Z-drugs, orexin receptor antagonists, and ramelteon—as well as monotherapy and concurrent use of multiple classes, with recurrent falls.

### 2.2. Data Collection and Variables

The patient characteristics assessed at the time of the index fall included age, sex, activity level, presence of dementia, and total number of medications. Based on the incident reports and medical records, activity level was classified into five categories: bedridden, requiring minimal assistance with mobility, walking with an assistive device, independent outdoor mobility, and no mobility impairment. The total number of medications was determined by reviewing the active prescription records in the electronic medical record at the date and time of the index fall. Only regularly scheduled oral medications that were active at that time were counted; medications that had been discontinued and as-needed medications were excluded.

Sleep-related medications were classified into four classes: (1) benzodiazepines used for insomnia or anxiety, (2) non-benzodiazepine hypnotics (Z-drugs), (3) orexin receptor antagonists, and (4) ramelteon. Benzodiazepines used for the treatment of seizures or epilepsy were not classified as benzodiazepines used for insomnia or anxiety.

Patients using any of these four medication classes were classified as users of sleep-related medications. Concurrent use of two or more of the four classes was defined as sleep-related medication polypharmacy. In addition, patients who were not using any other class of sleep-related medication were identified and classified into the following mutually exclusive monotherapy groups: benzodiazepine monotherapy, Z-drug monotherapy, orexin receptor antagonist monotherapy, and ramelteon monotherapy.

### 2.3. Statistical Analysis

All analyses were performed at the patient level using patient characteristics and medication use at the index fall. Patient characteristics and medication use were compared between single and recurrent fallers. Continuous variables are presented as means and standard deviations, whereas categorical variables are presented as numbers and percentages. Continuous variables were compared using the independent-samples *t*-test, and categorical variables were compared using the chi-square test.

Because recurrent falls occurred in 23.8% of the study population, odds ratios derived from logistic regression could overstate the magnitude of association when interpreted as risk ratios. Therefore, associations with recurrent faller status were examined using modified Poisson regression with a log link and a robust sandwich variance estimator, as described by Zou [16]. The results are presented as adjusted risk ratios (RRs) and 95% confidence intervals (CIs). In the drug-class-specific analysis, benzodiazepines used for insomnia or anxiety, Z-drugs, orexin receptor antagonists, and ramelteon were entered simultaneously into the same model. Model 1 was adjusted for age, sex, and activity level at the index fall. Model 2 was additionally adjusted for dementia and the total number of medications.

In separate regression models, we examined the associations of any sleep-related medication use and concurrent use of two or more sleep-related medication classes with recurrent falls. We also performed an analysis using mutually exclusive medication categories, with no sleep-related medication use as the reference category. These categories comprised benzodiazepine monotherapy, Z-drug monotherapy, orexin receptor antagonist monotherapy, ramelteon monotherapy, and concurrent use of two or more classes.

Patients with missing data for any variable included in a given model were excluded from that analysis. Statistical analyses were performed using IBM SPSS Statistics, version 28 (IBM Corp., Armonk, NY, USA). All tests were two-sided, and the overall significance level was set at 5%. For the four prespecified drug-class comparisons in the primary analysis, Bonferroni correction was applied, with statistical significance set at *p* < 0.0125. The mutually exclusive monotherapy analysis was considered exploratory.

### 2.4. Ethical Statements

This study was approved by the Ethics Committee of Niigata University (approval no. 2026-0167), date: 24 September 2026. Because of the retrospective observational design, individual written informed consent was not obtained. Information regarding the study was made publicly available, and participants were provided with the opportunity to opt out.

## 3. Results

### 3.1. Patient Characteristics

Among the 1830 patients included in the study, 1394 were classified as single fallers and 436 as recurrent fallers; recurrent fallers accounted for 23.8% of the study population (Table 1).

The mean age was 69.6 ± 14.4 years in the single-faller group and 69.2 ± 13.0 years in the recurrent-faller group, with no significant between-group difference. The single-faller and recurrent-faller groups included 748 and 250 men, respectively, with no significant difference in sex distribution. The mean total number of medications was significantly higher among recurrent fallers than among single fallers (7.6 ± 3.6 vs. 7.0 ± 3.6, respectively). The distribution of activity levels at the index fall also differed significantly between the two groups (Table 1).

### 3.2. Use of Sleep-Related Medications

Benzodiazepines for insomnia or anxiety were used by 111 single fallers (8.0%) and 62 recurrent fallers (14.2%). Z-drugs were used by 73 (5.2%) and 41 (9.4%) patients, orexin receptor antagonists by 241 (17.3%) and 115 (26.4%) patients, and ramelteon by 61 (4.4%) and 33 (7.6%) patients in the single- and recurrent-faller groups, respectively (Table 2).

Any sleep-related medication was used by 408 single fallers (29.3%) and 191 recurrent fallers (43.8%). Concurrent use of two or more sleep-related medication classes was observed in 73 single fallers (5.2%) and 57 recurrent fallers (13.1%) and was more frequent among recurrent fallers (Table 2).

### 3.3. Multivariable Analysis

In Model 1, the four classes of sleep-related medications were entered simultaneously, with adjustment for age, sex, and activity level at the index fall. Benzodiazepines used for insomnia or anxiety (adjusted RR, 1.56; 95% CI, 1.25–1.95; *p* < 0.001), Z-drugs (adjusted RR, 1.49; 95% CI, 1.13–1.95; *p* = 0.004), and orexin receptor antagonists (adjusted RR, 1.39; 95% CI, 1.16–1.66; *p* < 0.001) were associated with recurrent falls. Ramelteon was not significantly associated with recurrent falls (adjusted RR, 1.33; 95% CI, 1.00–1.78; *p* = 0.054).

In Model 2, which additionally adjusted for dementia and the total number of medications, benzodiazepines used for insomnia or anxiety (adjusted RR, 1.54; 95% CI, 1.22–1.94; *p* < 0.001), Z-drugs (adjusted RR, 1.46; 95% CI, 1.11–1.92; *p* = 0.006), and orexin receptor antagonists (adjusted RR, 1.37; 95% CI, 1.14–1.64; *p* < 0.001) remained associated with recurrent falls. In contrast, ramelteon was not significantly associated with recurrent falls (adjusted RR, 1.29; 95% CI, 0.96–1.74; *p* = 0.095) (Table 3).

Use of any sleep-related medication was associated with recurrent falls in both Model 1 (adjusted RR, 1.59; 95% CI, 1.35–1.87; *p* < 0.001) and Model 2 (adjusted RR, 1.53; 95% CI, 1.30–1.82; *p* < 0.001). Concurrent use of two or more classes also showed a strong association with recurrent falls in Model 1 (adjusted RR, 1.93; 95% CI, 1.56–2.39; *p* < 0.001) and Model 2 (adjusted RR, 1.83; 95% CI, 1.46–2.29; *p* < 0.001) (Table 3).

In the exploratory monotherapy analysis using no sleep-related medication use as the reference category and the covariates included in Model 2, benzodiazepine monotherapy was associated with recurrent falls (adjusted RR, 1.50; 95% CI, 1.11–2.04; *p* = 0.009), as was Z-drug monotherapy (adjusted RR, 1.89; 95% CI, 1.33–2.68; *p* < 0.001) (Table 4). Orexin receptor antagonist monotherapy showed a modest association with recurrent falls (adjusted RR, 1.27; 95% CI, 1.00–1.61; *p* = 0.048). However, this finding should be interpreted cautiously because the analysis was exploratory and the *p*-values were not adjusted for multiple comparisons. Ramelteon monotherapy was not significantly associated with recurrent falls (adjusted RR, 1.18; 95% CI, 0.67–2.07; *p* = 0.565). Concurrent use of two or more sleep-related medication classes was strongly associated with recurrent falls compared with no sleep-related medication use (adjusted RR, 2.09; 95% CI, 1.65–2.65; *p* < 0.001) (Table 4).

## 4. Discussion

In this study, we examined the association between sleep-related medication use at the index fall and recurrent falls among 1830 patients who experienced inpatient falls over a five-year period. Sleep-related medication use remained associated with recurrent falls after adjustment for age, sex, activity level, dementia, and the total number of medications. In particular, benzodiazepines used for insomnia or anxiety, Z-drugs, orexin receptor antagonists, and concurrent use of two or more sleep-related medication classes were associated with recurrent falls. Key previous studies relevant to the association between sleep-related medications and falls are summarized in Table 5.

Benzodiazepines may contribute to falls through sedation, muscle relaxation, impaired attention, and prolonged reaction time [10,15]. Rather than analyzing all benzodiazepine use as a single category, the present study specifically distinguished benzodiazepines used for insomnia or anxiety from those used for other indications. Nevertheless, their use remained associated with recurrent falls. This finding suggests that the necessity of benzodiazepine treatment should be reassessed after an initial fall, even when these medications are prescribed for insomnia or anxiety.

Although Z-drugs are chemically distinct from benzodiazepines, they exert sedative effects through gamma-aminobutyric acid type A receptors [17]. Z-drugs are sometimes perceived as safer than benzodiazepines; however, they may cause unsteadiness, amnesia, and impaired alertness during nocturnal awakenings. A systematic review demonstrated an increased risk of fractures associated with Z-drug use and also suggested increased risks of falls and injuries [18]. An association between insomnia medications, including Z-drugs, and inpatient falls has also been reported among hospitalized patients [19]. In the present study, Z-drug monotherapy was associated with recurrent falls even among patients who were not using any other class of sleep-related medication. This finding suggests that continued Z-drug treatment should be carefully reassessed after an initial fall, even when a Z-drug is used as monotherapy.

Orexin receptor antagonist use was associated with recurrent falls in the primary analysis in which all four sleep-related medication classes were entered simultaneously. In the exploratory monotherapy analysis, orexin receptor antagonist monotherapy showed a modest association with recurrent falls (adjusted RR, 1.27; 95% CI, 1.00–1.61; *p* = 0.048). However, the magnitude of this association was smaller than that observed in the primary analysis, and the result was borderline and unadjusted for multiple comparisons. Shishida et al. [20] reported that suvorexant did not significantly increase the risk of falls among patients admitted to a general hospital. Differences in the study population, outcome definition, and concomitant medication use may partly account for the differing findings. Therefore, the present exploratory result does not establish orexin receptor antagonist monotherapy as an independent risk factor for recurrent falls and should be interpreted cautiously. In addition, patients prescribed orexin receptor antagonists may have underlying characteristics related to medication selection, such as insomnia, cognitive impairment, or nocturnal agitation. Consequently, confounding by indication cannot be completely excluded [10,19].

Ramelteon was not significantly associated with recurrent falls in either the overall drug-class-specific analysis or the monotherapy analysis. Previous studies of hospitalized patients have similarly found no significant increase in fall risk associated with ramelteon use [19,20]. However, only 40 patients in the present study received ramelteon monotherapy, and the 95% CI was wide. Therefore, our findings do not establish that ramelteon is safer than other sleep-related medications, and the limited statistical power resulting from the small sample size should be considered.

The strongest association observed in this study was for the concurrent use of two or more classes of sleep-related medications. Compared with no sleep-related medication use, concurrent use was associated with an adjusted RR of 2.09 for recurrent falls. Overlapping sedative effects and next-day residual effects resulting from the concurrent use of multiple medications may have contributed to recurrent falls [13,14]. Conversely, patients requiring multiple medications may have more severe insomnia or may experience nocturnal agitation, delirium, or psychiatric symptoms [10,19]. Therefore, the observed association may reflect not only the additive effects of these medications but also the complexity of the patients’ underlying clinical conditions.

The clinical significance of this study lies in demonstrating the importance of medication review both at hospital admission and after an initial inpatient fall. STOPPFall and the World Guidelines for Falls Prevention and Management recommend the structured identification of fall-risk-increasing drugs (FRIDs) and reassessment of their necessity in older adults at risk of falling [14,21]. The American Geriatrics Society Beers Criteria also identify benzodiazepines and Z-drugs as medications requiring caution because of their association with falls and fractures in older adults [22]. Medication review should ideally begin at hospital admission to identify potentially modifiable fall-risk-increasing medications before a fall occurs. For patients who subsequently experience an inpatient fall, the necessity, indication, and concurrent use of sleep-related medications should be reassessed. In particular, when patients are using Z-drugs, benzodiazepines for insomnia or anxiety, or two or more sleep-related medication classes, consideration should be given to dose reduction, discontinuation, simplification to monotherapy, or substitution with nonpharmacological sleep interventions. However, because this was an observational study, our findings do not directly demonstrate that discontinuing these medications reduces recurrent falls.

The strengths of this study include the use of data from 2587 fall events reported over a five-year period and the inclusion of a relatively large population of 1830 patients. In addition, benzodiazepines were classified according to their indication, the four sleep-related medication classes were analyzed simultaneously, and monotherapy was distinguished from concurrent use of multiple classes.

### Limitations

Several limitations of this study should be acknowledged. First, this was a single-center retrospective observational study, and causality cannot be established. Second, because the study population included only patients who had experienced an inpatient fall, the association between sleep-related medication use and the occurrence of an initial fall could not be evaluated. Third, the severity of insomnia, delirium, nocturnal agitation, and psychiatric symptoms was not adequately adjusted for; therefore, residual confounding by indication may remain. Fourth, other medications acting on the central nervous system were not sufficiently adjusted for according to medication class, administration time, or actual administration status. Fifth, medication changes and fall-prevention interventions implemented after the index fall were not evaluated. Sixth, the length of hospitalization after the index fall and the observation period until the end of the study varied among patients, resulting in differences in the opportunity to experience a recurrent fall. Seventh, readmissions and differences among hospitalization episodes during the study period may have affected the classification of recurrent fallers. Eighth, most participants in this study overlapped with those included in a previous report [21]; therefore, the present findings do not represent replication in an independent cohort. Nevertheless, the present study addressed a different research question by newly evaluating the indications for sleep-related medication use, individual medication classes, monotherapy, and concurrent use of multiple classes. Future studies should include case–control studies involving patients without falls and prospective studies that treat length of hospitalization and medication changes as time-dependent variables.

## 5. Conclusions

Among patients who experienced inpatient falls, sleep-related medication use at the index fall was associated with recurrent inpatient falls after adjustment for age, sex, activity level, dementia, and the total number of medications. In particular, benzodiazepines used for insomnia or anxiety, Z-drugs, orexin receptor antagonists, and concurrent use of two or more sleep-related medication classes were associated with recurrent falls. The exploratory monotherapy analysis suggested a modest association for orexin receptor antagonist monotherapy; however, this finding should be interpreted cautiously because of multiple comparisons. Sleep-related medications should be reviewed at hospital admission and reassessed after an index inpatient fall, with particular attention to medication class, indication, and concurrent use of multiple classes.

## Figures and Tables

**Table 1:** Characteristics of single and recurrent fallers.

Characteristic	Single Fallers (*n* = 1394)	Recurrent Fallers (*n* = 436)	*p* Value
Age, years	69.6 ± 14.4	69.2 ± 13.0	0.586
Sex			0.196
Male	748 (53.7%)	250 (57.3%)	
Female	646 (46.3%)	186 (42.7%)	
Mobility status at the index fall			0.018
Bedridden	11 (0.8%)	2 (0.5%)	
Requiring minimal assistance with mobility	191 (13.7%)	76 (17.4%)	
Walking with an assistive device	773 (55.5%)	260 (59.6%)	
Independent outdoor mobility	369 (26.5%)	89 (20.4%)	
No mobility impairment	50 (3.6%)	9 (2.1%)	
Dementia	148 (10.6%)	59 (13.5%)	0.112
Total number of medications	7.0 ± 3.6	7.6 ± 3.6	0.006

Values are presented as mean ± standard deviation or number (%). Continuous variables were compared using the independent-samples *t*-test, and categorical variables were compared using the chi-square test.

**Table 2:** Use of sleep-related medications among single and recurrent fallers.

Sleep-Related Medication	Single Fallers (*n* = 1394)	Recurrent Fallers (*n* = 436)	*p* Value
Benzodiazepines for insomnia or anxiety	111 (8.0%)	62 (14.2%)	<0.001
Z-drugs	73 (5.2%)	41 (9.4%)	0.002
Orexin receptor antagonists	241 (17.3%)	115 (26.4%)	<0.001
Ramelteon	61 (4.4%)	33 (7.6%)	0.012
Any sleep-related medication	408 (29.3%)	191 (43.8%)	<0.001
Concurrent use of ≥2 classes	73 (5.2%)	57 (13.1%)	<0.001

Values are presented as numbers (%). Comparisons were performed using the chi-square test.

**Table 3:** Modified Poisson regression analyses of associations between sleep-related medication use and recurrent falls.

Exposure	Model 1 Adjusted RR (95% CI)	*p* Value	Model 2 Adjusted RR (95% CI)	*p* Value
Benzodiazepines for insomnia or anxiety	1.56 (1.25–1.95)	<0.001	1.54 (1.22–1.94)	<0.001
Z-drugs	1.49 (1.13–1.95)	0.004	1.46 (1.11–1.92)	0.006
Orexin receptor antagonists	1.39 (1.16–1.66)	<0.001	1.37 (1.14–1.64)	<0.001
Ramelteon	1.33 (1.00–1.78)	0.054	1.29 (0.96–1.74)	0.095
Any sleep-related medication	1.59 (1.35–1.87)	<0.001	1.53 (1.30–1.82)	<0.001
Concurrent use of ≥2 classes	1.93 (1.56–2.39)	<0.001	1.83 (1.46–2.29)	<0.001

RR, risk ratio; CI, confidence interval. Adjusted RRs and 95% CIs were estimated using modified Poisson regression with a log link and a robust sandwich variance estimator. Model 1 was adjusted for age, sex, and activity level at the index fall. Model 2 was additionally adjusted for dementia and the total number of medications. The four individual medication classes were entered simultaneously into the same model. Any sleep-related medication use and concurrent use of ≥2 classes were evaluated in separate models. For the four prespecified individual medication comparisons, statistical significance was defined as *p* < 0.0125 after the Bonferroni correction.

**Table 4:** Associations of sleep-related medication monotherapy and concurrent use of multiple classes with recurrent falls.

Medication Use	Single Fallers (*n* = 1394)	Recurrent Fallers (*n* = 436)	Adjusted RR (95% CI)	*p* Value
No sleep-related medication	986 (70.7%)	245 (56.2%)	1.00 (reference)	—
Benzodiazepine monotherapy	83 (6.0%)	36 (8.3%)	1.50 (1.11–2.04)	0.009
Z-drug monotherapy	38 (2.7%)	23 (5.3%)	1.89 (1.33–2.68)	<0.001
Orexin receptor antagonist monotherapy	184 (13.2%)	65 (14.9%)	1.27 (1.00–1.61)	0.048
Ramelteon monotherapy	30 (2.2%)	10 (2.3%)	1.18 (0.67–2.07)	0.565
Concurrent use of ≥2 classes	73 (5.2%)	57 (13.1%)	2.09 (1.65–2.65)	<0.001

Values are presented as numbers (%). RR, risk ratio; CI, confidence interval. Adjusted RRs and 95% CIs were estimated using modified Poisson regression with a log link and a robust sandwich variance estimator, with adjustment for age, sex, activity level at the index fall, dementia, and the total number of medications. The medication categories were mutually exclusive, and no sleep-related medication use was the reference category. This analysis was exploratory, and the *p*-values were not adjusted for multiple comparisons. Unrounded estimates were used to calculate *p* values; therefore, the rounded lower confidence limit for orexin receptor antagonist monotherapy is displayed as 1.00 despite *p* = 0.048.

**Table 5:** Summary of key previous studies on sleep-related medications and falls.

Study	Study Design and Population	Medication(s) Evaluated	Outcome	Principal Finding
Seppala et al. [10]	Systematic review and meta-analysis	Fall-risk-increasing drugs, including psychotropic medications	Falls	Certain psychotropic medications were associated with an increased risk of falls.
Treves et al. [16]	Systematic review and meta-analysis	Z-drugs	Falls, fractures, and injuries	Z-drug use was associated with an increased risk of fractures, with possible increases in falls and injuries.
Herzig et al. [17]	Retrospective cohort study of hospitalized patients	Medications used for insomnia	In-hospital falls	Several medications commonly used for insomnia were associated with in-hospital falls.
Shishida et al. [18]	Observational study of hospitalized patients	Suvorexant and ramelteon	In-hospital falls	Suvorexant and ramelteon were not associated with a significant increase in fall risk.
Present study	Single-center retrospective observational study of 1830 patients with inpatient falls	Benzodiazepines, Z-drugs, orexin receptor antagonists, and ramelteon	Recurrent inpatient falls	Benzodiazepines, Z-drugs, orexin receptor antagonists, and concurrent use of ≥2 classes were associated with recurrent falls. In the exploratory analysis, orexin receptor antagonist monotherapy showed a modest association, whereas ramelteon monotherapy was not significantly associated.

## Data Availability

The data presented in this study are available on reasonable request from the corresponding author. The data are not publicly available due to ethical restrictions and the protection of participant privacy.

## Abbreviations

The following abbreviations are used in this manuscript:

AGS	American Geriatrics Society;
CI	confidence interval;
FRIDs	fall-risk-increasing drugs;
RR	risk ratio;
SPSS	Statistical Package for the Social Sciences;
STOPPFall	Screening Tool of Older Persons Prescriptions in older adults with high fall risk
